# Laser direct structuring and electroless plating applicable super-engineering plastic PPS based thermal conductive composite with particle surface modification

**DOI:** 10.1039/c8ra00967h

**Published:** 2018-03-12

**Authors:** Kiho Kim, Jinseong Lee, Seokgyu Ryu, Jooheon Kim

**Affiliations:** School of Chemical Engineering & Materials Science, Chung-Ang University Seoul 06974 Republic of Korea jooheonkim@cau.ac.kr; DREAMTECH Co., Ltd Gunpo 15849 Republic of Korea

## Abstract

Boron nitride (BN) and laser activate particles (LAPs) were surface-modified *via* base treatment and by using a silane coupling agent in order to confer functionality and enhance the interfacial affinity of these particles for a polymer matrix. The introduction of LAP and BN caused severe deterioration of the mechanical properties of the filler–polymer composite by acting as defects and due to the poor interface with polyphenylene sulfide (PPS), used as the polymeric matrix. As expected, the thermal and mechanical properties were enhanced *via* surface modification, whereas the tensile strength of the composites with the surface-modified fillers remained lower than that of neat PPS. The BN/LAP binary filler system showed little influence on the mechanical properties of the composite. However, the incorporation of a small amount of LAP into the BN composite produced a slight improvement of the thermal conductivity when the total filler content was maintained. Moreover, LAP leads the metal plating at the laser irradiated surface. Thus, the BN/LAP/PPS composite was used to fabricate a circuit board *via* laser direct structuring (LDS) and electroless plating for potential light emitting diode (LED) application.

## Abstract 2

Boron nitride (BN) and laser activate particles (LAPs) which used for electroless plating were surface-modified *via* base treatment and by using a silane coupling agent in order to confer functionality and enhance the interfacial affinity of these particles for a polymer matrix.

## Graphical abstract

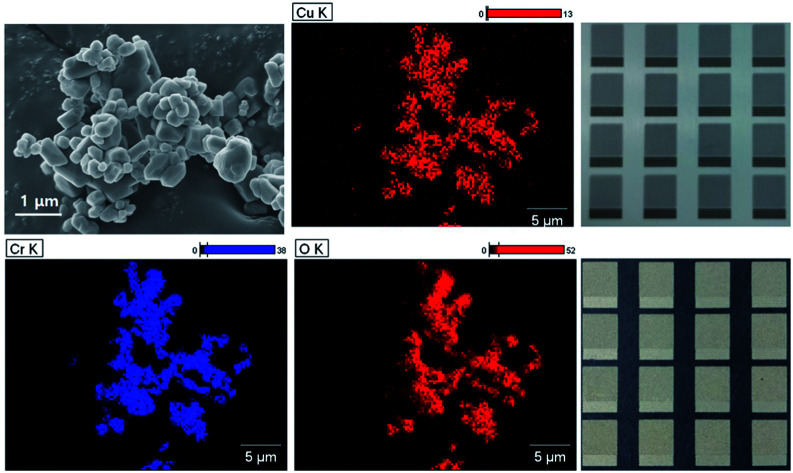

## Introduction

Thermally conductive polymeric composites have attracted much attention because of their broad applications in electronic, high-temperature dielectric, and energy storage devices.^1^ Particularly, electronic gadgets require increasingly high thermal conductivity to achieve high integrity, performance, and multi-functionalization.^2^ It is well known that a high device temperature considerably decreases the reliability and lifespan; thus, generated heat should be removed from the device by using a highly thermally conductive composite.^3^ Many kinds of thermally conductive composites have been used in electronic devices, such as printed circuit boards (PCBs), package substrates, thermal interface materials (TIMs), housing materials, thermal greases, and so on. TIMs and thermal grease based on thermoset and siloxane resins with ceramic fillers are used to reduce the interface resistance. Housing materials have been fabricated *via* injection molding of thermoplastics, where the emissivity is important for releasing heat from the device into the atmosphere. Thermoset-based PCBs and package substrates require the highest thermal conductivity among the aforementioned applications, as the heat sources are directly mounded. In addition, phase change material (PCMs) such as nanofluids with thermally conductive particles, have been studied for thermal management, but have found minimal application in highly integrated devices due to their size.^4^

Recently, thermoplastic-based PCBs have been widely researched due to their good mechanical properties and processability. Notably, ultra-high performance thermoplastics with thermal resistivity, such as polyphenylene sulfide (PPS), liquid crystalline polymers (LCPs), and polyetherimide (PEI), have been classified as super-engineering plastics (SEPs), and have been applied to PCBs and various housing materials.^5^ The most important advantage of SEP-based PCBs is that they are free from structural dimensions; spherical and cylindrical PCBs are realizable, whereas traditional thermoset-based PCBs can only be fabricated in 2D shapes. The 3D circuit carriers offer enormous potential for enhancement of the functionality and simultaneous miniaturization of the overall size of the electronic systems. These 3D-molded interconnect devices (3D-MID) are manufactured by injection molding and structuring of 3D circuitry. However, the general circuit printing method is limited to 2D substrates and requires a complex process. The photo-imaging technique is extensively used in flat circuit pattering, but can also be used for circuit pattering in MIDs using a 3D mask; it also requires many steps such as masking, deposition, and etching, some of which emit environmentally hazardous chemicals.^6^

The circuit printing problems originating from the high-dimensional structures and printing process can be overcome *via* laser direct structuring (LDS) and electroless plating. LDS makes it possible to substitute traditional circuit boards in mechatronics assemblies. The structures of the conductive paths are written onto the plastic with a laser, and physical–chemical reaction forms metallic nuclei that act as a catalyst for reductive copper plating. In addition to activation, the laser creates a microscopically rough surface in which the copper is firmly anchored during metallization. Moreover, electroless plating is a non-galvanic plating method that involves several simultaneous reactions in an aqueous solution; these occur without the use of external electrical power. This results not only in noticeable weight savings, but also in a significant reduction in costs due to installation advantages.^7^

In this study, a PPS-based thermally conductive composite is fabricated for 3D-MIDs by using surface-modified BN and LAP fillers. PPS is a suitable polymeric thermoplastic because LDS requires the compound to have good heat resistance, and above all, to be highly suitable for metallization. In this system, LAP dispersion influences the plating efficiency. Moreover, high particle dispersion and strong interfacial affinity are very important factors for achieving high thermal conductivity and mechanical properties. Therefore, BN and LAP were modified *via* base treatment and with a silane coupling agent. Silane coupling agents are the most widely used surface modifying agents because of their good reactivity with both organic and inorganic materials. Finally, the thermal and mechanical properties are investigated by variation of the filler content and composition in order to fabricate LDS and electroless platable highly thermally conductive PCBs.

## Experimental

### Particle surface modification

Firstly, the surface of LAP was treated with sodium hydroxide solution for hydroxyl group functionalization. LAP (10 g) was suspended in 5 M NaOH solution at 80 °C for 12 h and then rinsed with deionized (DI) water and filtered several times to restore the pH from basic to neutral. Secondly, the hydroxyl-functionalized LAP was modified by using silane coupling agents: (3-aminopropyl)triethoxysilane (APTES). APTES (3 wt%) was added to deionized (DI) water and ethanol (7 : 3 mixture) and stirred at 50 °C for 30 min to achieve hydrolysis. The as-prepared particles (10 g) and oxalic acid (as a catalyst) were added to the above solution and stirred at 80 °C for 12 h. The resulting particles were rinsed with DI water, filtered three times, and then dried in a convection oven at 80 °C for 5 h to remove the solvent.

BN surface modification was performed by a similar method with same chemical solutions. In the case of BN, the reactions were performed for 48 h and 24 h with 5 M NaOH and APTES solution at 120 °C and 80 °C, respectively. The resultant LAP and BN particles are denoted as LAP-NH2 and BN-NH2, respectively.^8^

### Fabrication of composite

The PPS based composite materials with various ratios of LAP and BN particles were prepared *via* the melt-mixing method using a twin extruder (model BA-11, *L*/*D* ratio = 40; Bau Technology) in a specified temperature range. The temperature of the feeding zone, melting zone, mixing zone, and exit die were 270, 280, 290, and 300 °C, respectively. The feeding rate of the materials and the blade speed were maintained constant at 50 g min^−1^ and 200 rpm, respectively. The melt-mixed composites were immediately quenched in a water-bath after extrusion. The composites were pelleted and dried in a convection oven at 80 °C for one day before use. Specimens for the mechanical and thermal property tests were prepared using a mini injection molder (DSM Xplore, Micro Injection Moulding Machine, 5.5 mL).

### Characterization

Surface modification of LAP was confirmed by X-ray photoelectron spectroscopy (XPS, Thermo U.K. K-Alpha) using an Al-K_α_ X-ray source (1486.6 eV) and a hemispherical analyzer. During curve fitting, the Gaussian peak widths in each spectrum were constant. Thermogravimetric analyses (TGA; TGA-2050, TA Instruments) of the samples were carried out to examine the thermal degradation process. The samples (4 mg) were heated to 800 °C at a heating rate of 10 °C min^−1^ under nitrogen atmosphere. Field emission scanning electron microscopy (FE-SEM, Sigma, Carl Zeiss) was used to examine the morphology of the particles and fabricated composites. The thermal transport performance of the fabricated composites was characterized by laser flash analysis (LFA, Netzsch Instruments Co, Nanoflash LFA447) and differential scanning calorimetry (DSC, Perkin-Elmer Inc., DSC-7) at room temperature. The transferred signal initiated a thermal equilibration process in the composite specimen, which was recorded by using a difference detector at the rear surface and was used to evaluate the thermal diffusivity. The bulk density (*ρ*_comp_ (g cm^−3^)) of the specimens was measured by using the Archimedes water displacement method. The thermal conductivity (*k*) was calculated by multiplying the thermal diffusivity, density, and specific heat capacity of the composite. Tensile tests were performed with a universal testing machine (UTM, R&B Corp, model UTM-301) at a crosshead speed of 5 mm min^−1^. The storage modulus of the composite was measured by using dynamic mechanical analysis (DMA; Triton Instrument, Triton DMTA). The storage modulus of the composite were measured at a frequency of 1 Hz and a heating rate of 5 °C min^−1^ according to ASTM1640 and analyzed in the tensile mode.

**Fig. 1:**
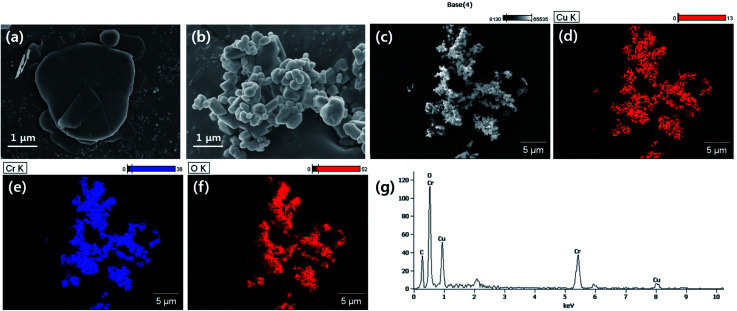
Morphological analysis of used fillers. (a) BN, (b) LAP, (c)–(f) EDS atomic analysis of LAP (g) EDS spectrum of LAP.

## Results and discussion

**Fig. 2:**
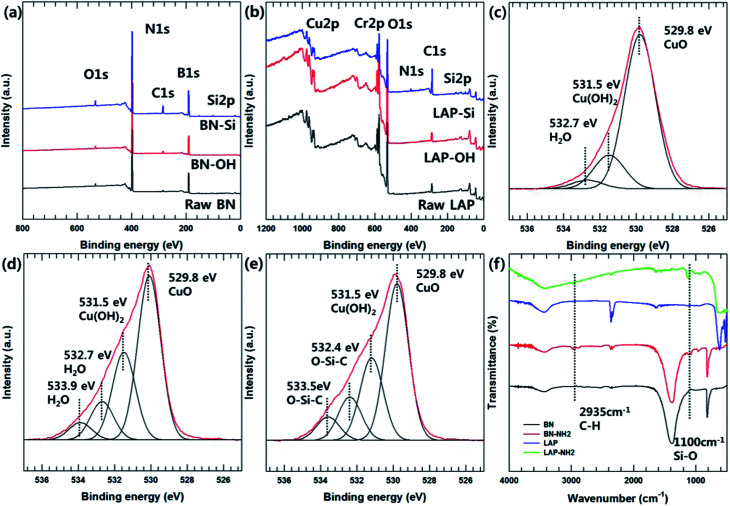
XPS and FT-IR analysis of particle surface modification. (a) XPS survey scan spectra of BN and surface modified BN, (b) XPS survey scan spectra of LAP and surface modified LAP, (c)–(e) O1s spectra of raw LAP, LAP-OH, and LAP–Si, (f) FT-IR analysis.

The particle morphology was observed by FE-SEM and EDS (Fig. 1). It is well known that BN has a 2-dimensional hexagonal shape with a smooth surface. LAP has an irregular shape with sub-micron dimensions, and is an alloy of copper and chrome oxide as shown in EDS analysis. Surface modification of LAP and BN was confirmed by XPS and FT-IR analysis (Fig. 2). Fig. 2(a) shows the XPS wide scan spectra of BN and surface-modified BN. Two strong peaks were observed around 191 and 398 eV, indicative of boron and nitrogen species in raw BN, whereas an oxygen peak emerged at 533 eV after sodium hydroxide treatment. Moreover, a silicon peaks appeared at 101 and 151 eV in the XPS profile of BN–Si, providing evidence of introduction of the silane coupling agent.^9^ The preparation of silane-coupling modified BN particles was reported is several of our previous studies, thus a detailed analysis is not presented in this manuscript. Fig. 2(b)–(e) shows the XPS data for surface-functionalized LAP. In the wide-scan spectra, raw LAP and LAP-OH show the same peaks because raw LAP has a sufficient amount of oxygen atoms. However, the O1s spectra in Fig. 2(c) show a notable change after base treatment; the intensity of a copper hydroxide peak increased at 531.5 eV, whereas the profile of raw LAP showed only peaks, strongly suggesting that hydroxyl groups were introduced onto the surface of LAP by base treatment. Moreover, the generated O–Si peaks ant 532.4 and 533.5 eV were strong evidence of successful surface modification *via* NaOH and APTES treatment in Fig. 2(e).^10^ The weak Si and N peaks in survey scan spectra that emerged after treatment with the silane coupling agent also provide further evidence of surface modification, as these peaks were absent in the profile of raw LAP. Unfortunately, the intensity of these peaks was too weak as the amount of silane coupling agent was too small, thus, detailed analysis could not be performed using Si and N. Fig. 2(f) provide more evidence for surface modification of BN and LAP *via* APTES, the absorption peaks of Si–O and C–H bonds were appeared at 1100, and 2935 cm^−1^ on the both APTES treated particles. From these results, it could confirm that BN and LAP were modified *via* base and APTES treatments.^11^

**Fig. 3:**
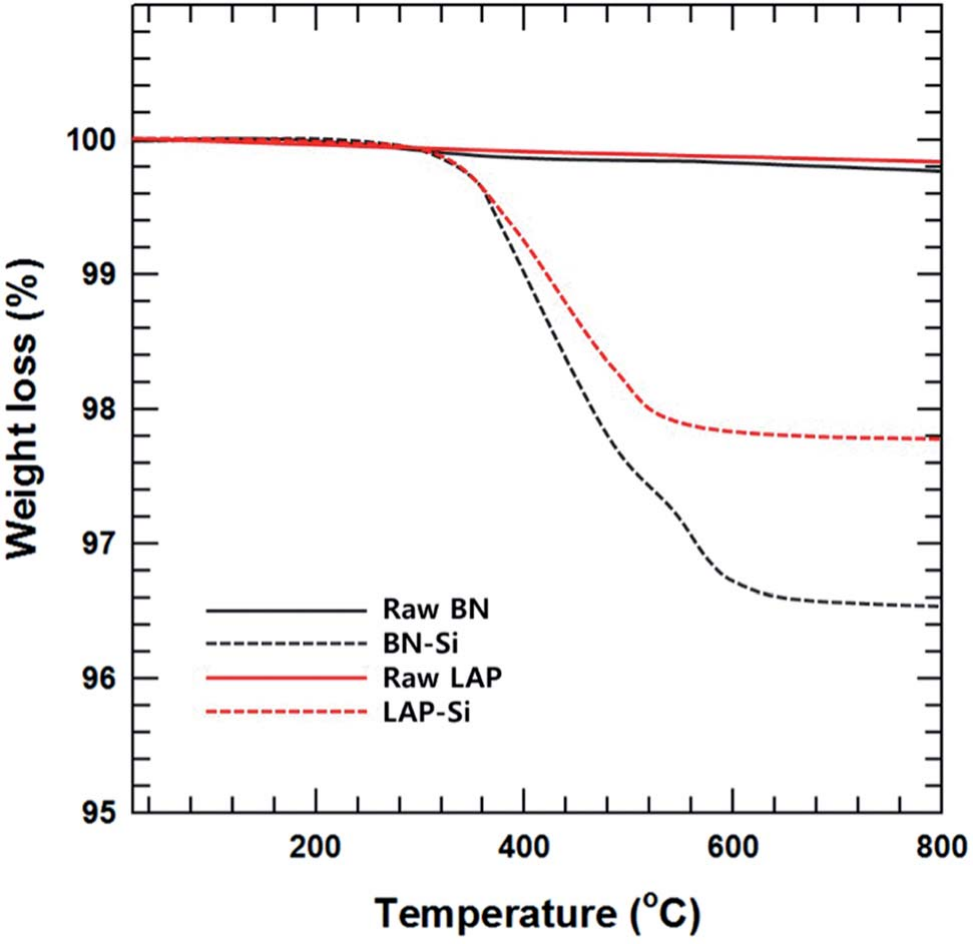
TGA results of surface modified BN and LAP using silane coupling agents.

Thermogravimetric analysis (TGA) was performed to quantitatively assess the surface modification of BN and LAP with the silane coupling agent. Almost no weight loss was observed for raw BN and LAP under the experimental conditions due to the heat resistivity; BN and most metal oxides require extremely high temperatures for thermal degradation. On the contrary, the surface-modified particles underwent slight thermal degradation around 3.5 and 2.2% weight loss at 400 to 600 °C, caused by degradation of the silane coupling agent (Fig. 3).

**Fig. 4:**
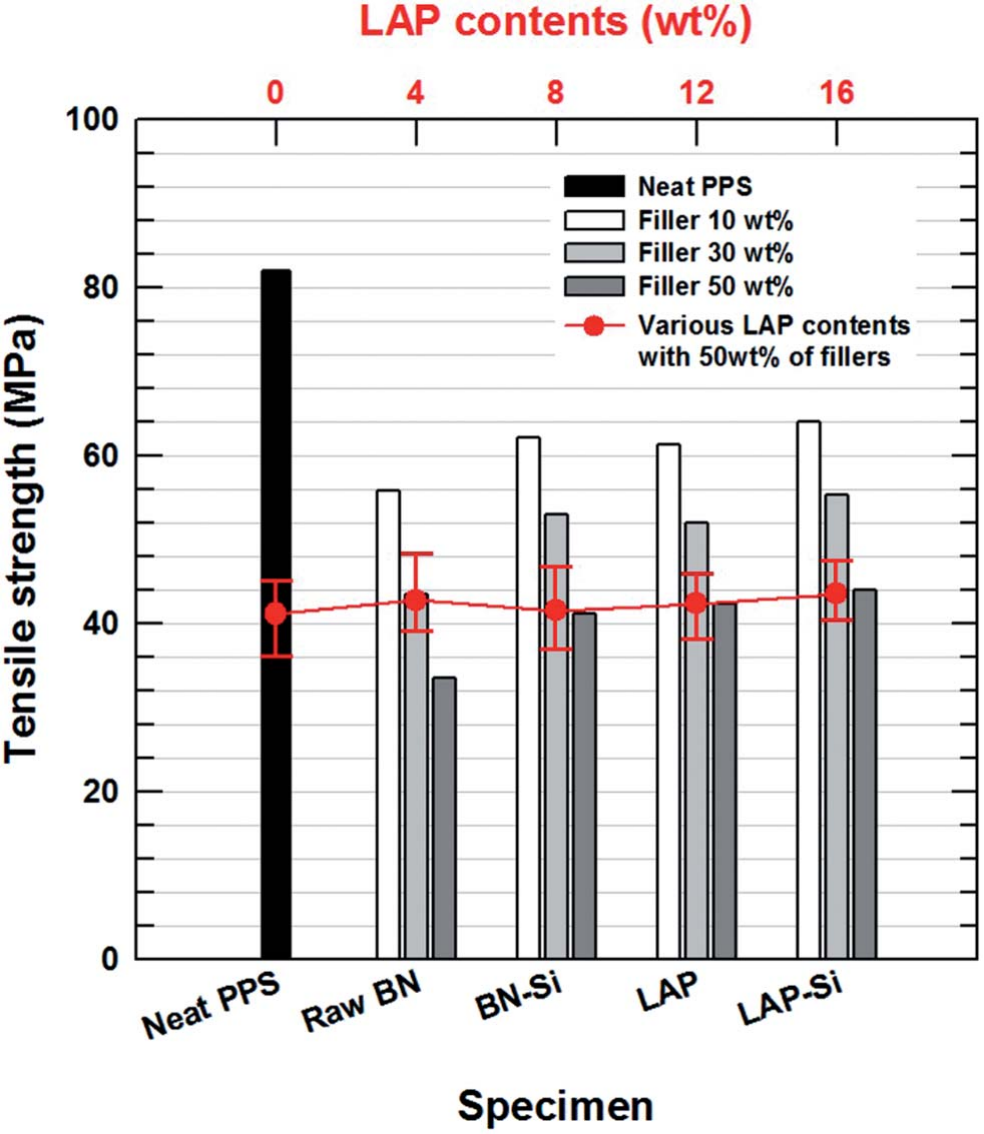
Tensile strength of neat PPS and composites with various filler concentrations. The red line and scatter plot indicate the tensile strength of surface modified filler composite with various LAP contents and fixed total filler contents to 50 wt%.

The mechanical strength was examined by using a UTM. As shown in Fig. 4, the presence of BN and LAP in the PPS matrix significantly reduced the tensile strength of the composite, as the metal oxide particles act as an impurity between the polymer chains. Moreover, the absence of strong functional groups on LAP and the weak interaction between the polymer matrix (attributed to micron-size clusters of agglomerated particles), and the presence of voids in the composites, which introduced more concentrated stresses on the interface, resulted in a decrease in the tensile strength. Similarly, the micron-scale ceramic particles also act as serious defects.^12^ BN is a well-known unfriendly ceramic material due to its repulsive interaction with polymeric materials. After surface modification, however, the stress concentration was lower and the stresses could be more easily transferred from the matrix to the particles than in the case of the raw LAP and BN composites, whereas the tensile strength decreased relative to that of neat PPS (Table 1). Intimate contact between the particles and the matrix also ensured a reduction of crack propagation. Furthermore, the mixing composition of the two particles had almost no effect on the tensile strength because both particles play the same role without particular interaction.

**Fig. 5:**
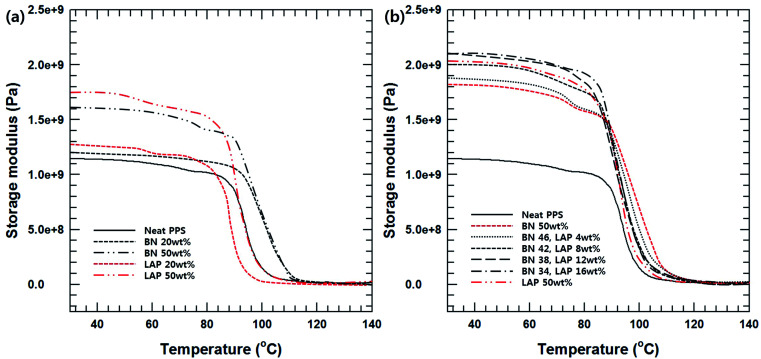
(a) Storage modulus of BN and LAP/PPS composite with various filler contents, (b) storage modulus of surface modified BN and LAP/PPS composite with various filler composition.

The dynamic mechanical properties of the BN/LAP/PPS composites were verified by variation of the filler content, surface modification and composition. DMA measurement is effective for estimating the interfacial interaction between the reinforcement particles and the matrix. As shown in Fig. 5, particle loading and surface modification led to a notable enhancement of the storage modulus. This could be attributed to the better stress transfer from the matrix to the included reinforcement particles, which was operative mainly at temperatures lower than the glass transition point. Moreover, the higher storage modulus of the LAP composite relative to those of the BN composite can be attributed to the strong mutual interaction between LAP and PPS, which decreases the interfacial slide and relaxation. This phenomenon ultimately results in decreased lag, thereby lowering the tan *δ* value.^13^ The interfacial interaction was strong because of the good dispersion, and the increase in the surface area and surface energy provided more efficient interfacial bonds between the filler and PPS on the nanoscale. Moreover, nanoscale LAP can function as pseudo-crosslinking points, which results in a marked increase in the storage modulus of the PPS composites containing LAP relative to the micron-scaled BNs at the same filler content. Notably, the BN/LAP binary system showed a higher modulus than the BN and LAP composites, which differed from the trends in the tensile strength. Moreover, a small amount of LAP enhanced the modulus of the BN/PPS composite relative to that of the LAP composite with added BN. These results are also related to the dispersion of the nanosized filler. As previously mentioned, dispersion of the nano-filler without aggregation has a strong effect on the modulus. Previous studies reported that the rotation of large particles during the compounding process causes a shear force that mechanically disperses the smaller aggregated particles, similar to stirring.^14^ Dispersion of the nanoparticles between the large BN particles also effectively transfers the stress. On the other hand, fewer large particles did not generate sufficient shear force, where the agitating effect is very weak, thereby curtailing the synergetic effect.

**Fig. 6:**
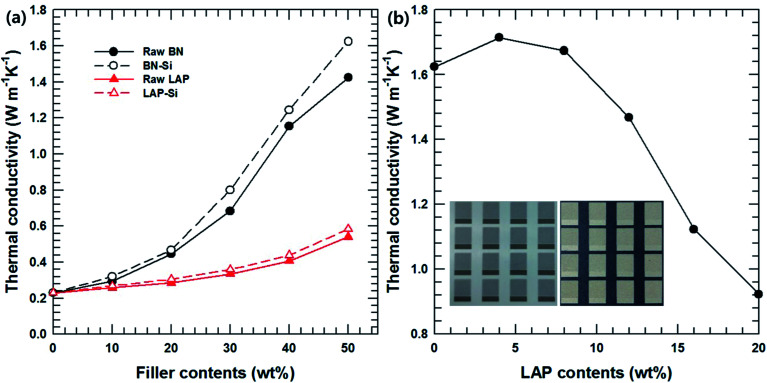
Thermal conductivities of composites. (a) The function of filler contents and surface modification, (b) the function of surface modified LAP contents with fixed total filler contents to 50 wt%. The inserted picture is BN/PPS (left) and BN/LAP/PPS (right) composite after the LDS and Cu/Ni electroless platting.

The thermal conductivity of the BN/LAP/PPS composite was examined to evaluate the particle surface modification and filler composition. Fig. 6(a) shows the effect of surface modification of the BN and LAP composites as a function of the filler content. It was expected that the thermal conductivities of all composites would increase continuously with increasing filler content, which is the general behavior, because heat flow paths are more easily generated at higher filler contents. Notably, the BN composite showed outstanding thermal conductivity relative to the LAP composite because BN is a widely used thermally conductive filler with outstanding performance (thermal conductivity above 300 W m^−1^ k^−1^). Unfortunately, the thermal conductivity of LAP has not been reported as it is specially used as an additive for LDS and electroless plating. It appears that LAP has lower thermal conductivity than BN based on the above results, although the value is unknown. However, most metal oxides have relatively high thermal conductivity (around 10 W m^−1^ k^−1^); the thermal conductivity of the composite could also be controlled by varying the LAP content. Moreover, the thermal conductivity of both composites was enhanced *via* particle surface modification; these results are consistent with the mechanical properties. Fig. 6(b) presents the thermal conductivity of the composites with a mixture of surface-modified BN and LAP with a fixed total filler content of 50 wt%. With an increase in the LAP content, the thermal conductivity decreased due to the relatively lower thermal conductivity of LAP. However, 4 wt% and 8 wt% of LAP caused a slight increase relative to the thermal conductivity of the BN composite. These results are notable because they indicate a synergetic effect of the fillers on the thermal conductivity. Moreover, a certain amount of LAP is required for electroless plating. As shown in the inset, the BN composite was not effective for building a copper/nickel circuit, whereas 4 wt% LAP was effective for developing the metal layer. Therefore, the small amount of LAP particles not only acted a metallic seed, but also as a thermally conductive filler for MIDs.

**Fig. 7:**
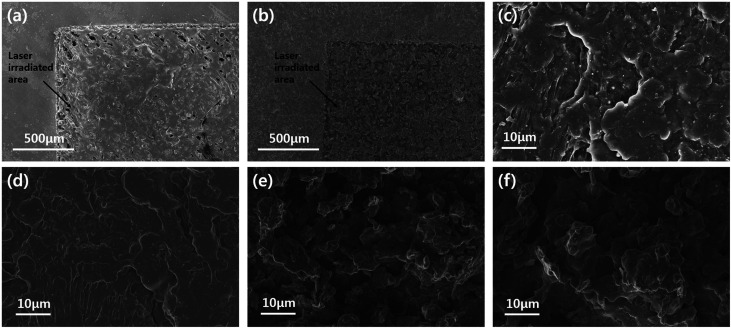
FE-SEM top-view images. (a) Neat PPS, (b) and (c) raw BN/LAP/PPS, (d) BN–Si/LAP–Si/PPS composite, (e) and (f) raw BN/LAP/PPS and BN–Si/LAP–Si/PPS of laser irradiated surface.

**Fig. 8:**
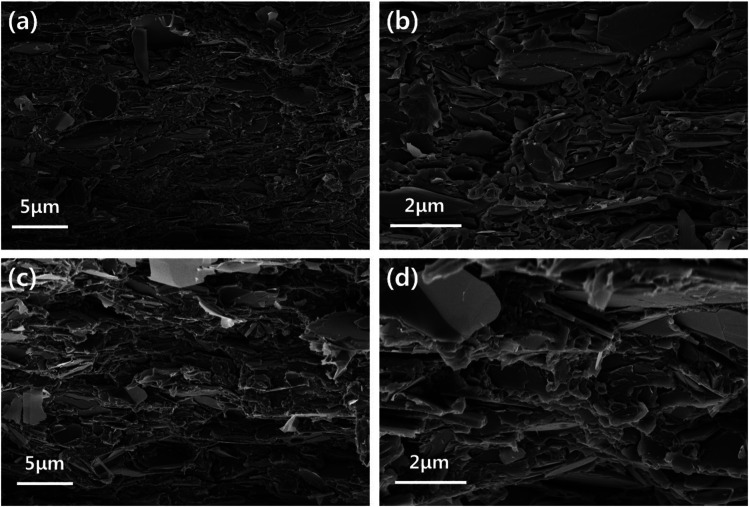
FE-SEM cross-sectional images. (a) and (b) Low and high magnification images of raw BN/LAP/PPS, (c) and (d) low and high magnification images of BN–Si/LAP–Si/PPS.

In order to confirm the dispersion of the particles, the top-view and cross-sectional images of the BN/LAP/PPS composite were observed *via* FE-SEM. Fig. 7(a) and (b) shown the top-view images of laser patterned neat PPS and surface modified BN/LAP/PPS composites after LDS *via* laser irradiation. Regardless of BN and LAP particles, both composites were obviously patterned *via* laser irradiation because laser degrade the polymeric materials and make the trace. From the top-view images, the surface modification effect were clearly observed, pristine particles were protrude at the surface while surface modified particles were smoothly covered to PPS matrix. Those protrude LAPs could cause the metal plating at the both patterned and unwanted other surfaces. Unfortunately, the definite difference *via* particle surface modification did not confirmed at the laser radiated surface due to covered polymeric materials were etched at the both composite. In order to confirm the distinguishable difference cross-sectional images were shown in Fig. 8. As shown in Fig. 8(a) and (b), raw BN and LAP showed poor interfacial affinity due to the absence of specific interactions with the PPS matrix. Specifically, BN produced an extremely poor interface, where many air voids were observed between the basal and matrix components. However, the modified interface was easily observed after particle surface treatment with the silane coupling agent, those results were clearly support the mechanical and thermal properties of composites.

**Table 1:** Mechanical properties of neat PPS and their composites. (The values in the parenthesis were error range)

	Tensile strength [MPa]	Young's modulus [MPa]	Failure strain [%]
Neat PPS	82.6 (4.1)	1679.8 (32.1)	56.6 (21.1)
Raw BN, 50 wt%	33.5 (4.6)	1856.3 (51.3)	16.3 (3.1)
BN–Si, 50 wt%	41.2 (2.7)	2048.2 (40.4)	13.4 (2.2)
Raw LAP, 50 wt%	42.3 (3.3)	1926.7 (44.1)	22.8 (5.5)
LAP–Si, 50 wt%	44.1 (2.8)	2125.4 (28.6)	14.7 (3.7)

## Conclusion

For 3D-MID application, thermally conductive composites based on the LDS-applicable super-engineering plastic PPS were fabricated by using the thermally conductive fillers BN and LAP. The interfacial affinity between the polymeric matrix and the filler particles greatly influenced the mechanical properties and thermal conductivity. However, BN and LAP have poor affinity for the PPS matrix, and not only caused the mechanical properties to deteriorate, but also showed low thermal conductivity. Therefore, BN and LAP were chemically modified by using NaOH solution and silane coupling agent, APTES. APTES treatment effectively enhance the particle/polymer interaction resulting in it prevents the degradation of the mechanical properties and enhances the thermal conductivity of the composites. Moreover, for the BN/LAP/PPS binary filler composite, there are no consistent synergetic effect of the particles on the mechanical properties, whereas a slight enhancement of the thermal conductivity was observed despite the lower BN content. After laser irradiation and plating, the composites with 4 wt% of LAP contents were successfully used to print a copper/nickel circuit.

## Conflicts of interest

There are no conflicts to declare.

## Supplementary Material
